# A simplified geometric CMR method for reproducible quantification of left ventricular thrombus volume: Left Ventricular thrombus: a standardized measurement method

**DOI:** 10.1007/s10554-026-03752-3

**Published:** 2026-06-15

**Authors:** Antonin Fuzeau, Denis Angoulvant, Julien Pucheux, Nicolas Cazeneuve, Claire Bouleti, Loïc Biere, Didier Bresson, Ahmad Hayek, Charles De Bourguignon, Cyrille Bergerot, Naoual El Jonhy, Simon Leboube, Marine Tortigue, Benoit Lattuca, François Roubille, Nathan Mewton, Pierre Croisille

**Affiliations:** 1 Cardiology, Centre Hospitalier Universitaire Trousseau, Tours, France; 2 Radiology, Centre Hospitalier Universitaire Trousseau, Tours, France; 3 ISCHEMIA Membrane Signalling and Inflammation in Reperfusion Injuries Cardiology, UMR Université de Tours/Inserm, University of Poitiers, Tours, 1327 France; 4 Clinical Investigation Center (CIC) INSERM 1402, Poitiers University Hospital, Poitiers, France; 5 Department of Cardiology and SFR ICAT, University Hospital of Angers, Angers, F-49000 France; 6 University Hospital of Mulhouse, Hôpital Emile Muller, Mulhouse, France; 7 Cardiology, Clinical Investigation Center Inserm 1407, Hospices Civils de Lyon, Université Claude Bernard Lyon 1, Lyon, Auvergne-Rhône-Alpes, France; 8 Cardiopaediatrics, Hospices Civils de Lyon, Auvergne-Rhône-Alpes, France; 9 ACTION Study Group, Cardiology Department, Nîmes University Hospital, Montpellier University, Nîmes, France; 10 ACTION Study Group, Cardiology Department, Montpellier University Hospital, Montpellier University, Montpellier, France; 11 Univ Lyon, CREATIS, UJM-Saint-Etienne, INSA, CNRS UMR 5520, INSERM U1294, Saint-Etienne F-42023, Saint-Etienne, France; 12 Radiology Department, University Hospital of Saint-Etienne, Saint-Etienne, France

**Keywords:** Left ventricular thrombus, STEMI, Cardiac magnetic resonance, Cardiovascular imaging

## Abstract

Left ventricular thrombus (LVT) is a frequent complication after myocardial infarction and is associated with increased risk of systemic embolism and adverse cardiovascular outcomes. However, the relationship between thrombus characteristics and its clinical evolution remains poorly understood, partly due to the lack of standardized and reproducible measurement methods. This study evaluated the feasibility and reproducibility of a simplified geometric CMR approach for standardized LVT volume assessment. This post-hoc analysis included patients from the COVERT-MI trial who underwent cardiac magnetic resonance (CMR) imaging 5 days after reperfusion for ST-segment elevation myocardial infarction. LVT volume was estimated using a simplified geometric method based on three orthogonal measurements (ABC/2 formula). Thrombus morphology, surface area, and insertion circumference were also assessed. Inter- and intra-reader reproducibility were evaluated using Bland–Altman analysis and Cohen’s kappa coefficients. Associations with thrombus persistence at 3 months and clinical outcomes at 1 year were explored as secondary analyses. Among 161 patients who underwent baseline CMR, 25 (15.5%) had LVT. The mean estimated thrombus volume was 1.03 ± 1.09 cm³. At 3 months, thrombus regression occurred in 80% of patients. The ABC/2 method showed high reproducibility. Inter-observer reproducibility demonstrated a bias of 0.07 cm³, whereas intra-observer reproducibility showed a bias of 0.03 cm³. Inter-observer correlation was high (*r* = 0.86, SEE = 0.51 cm³), while intra-observer correlation was excellent (*r* = 0.99, SEE = 0.15 cm³). Accounting for thrombus shape had minimal impact on volume estimation (mean difference 0.035 cm³). No thrombus characteristic was significantly associated with thrombus persistence, and no cardioembolic events occurred during the 1-year follow-up. A simplified geometric CMR approach based on the ABC/2 formula provides a feasible and highly reproducible framework for estimating LVT volume. Further studies in larger cohorts are needed to determine the clinical and prognostic relevance of quantitative thrombus assessment.

## Introduction

Left ventricular thrombus (LVT) is a severe complication following myocardial infarction, associated with prolonged hospital stays, increased healthcare costs, and systemic embolization, including cerebral stroke (occurring in up to 15% of patients with LVT)[1–13]. Despite its clinical significance, LVT is frequently underdiagnosed by transthoracic echocardiography (TTE), with a sensitivity of only 29% compared to 88% for cardiac magnetic resonance (CMR)[12, 14–19]. The prevalence of LVT remains substantial, affecting up to 12% of ST-elevated myocardial infarction (STEMI) patients when assessed by CMR[7, 9].

LVT is a major source of morbidity and mortality. Post-STEMI patients with LVT face a higher risk of major adverse cardiovascular events (MACE), mortality [20], short-term cerebrovascular events[3, 4, 8], and systemic thromboembolism. It is estimated that the presence of LVT increases embolic events by fourfold[20]. There is very limited data on this thromboembolic risk in the long term[21], but in retrospective registries LVT regression was associated with reduced mortality[22].

Prior CMR studies have already quantified LVT volume by planimetry and have explored its association with thrombus detection, persistence, and embolic outcomes. However, planimetric quantification is relatively time-consuming, reader-dependent, and not easily harmonized across centers or software platforms.[12, 21, 23–25].

Recent expert recommendations[26, 27] highlight the lack of solid evidence and the challenges in monitoring and treating LVTs. Management of LVT remains highly heterogeneous regarding the optimal antithrombotic therapy choice and duration and the timing of initial and follow-up imaging are not clearly defined.

Current therapeutic strategies for LVT do not consider thrombus shape or volume, despite evidence suggesting that initial thrombus area may influence regression [22].

Although CMR planimetry has been used to quantify LVT burden, a simple, prespecified, and easily transferable framework for standardized geometric assessment has not been established.

Before LVT volume or shape can be meaningfully integrated into clinical decision-making, a robust, reproducible and standardized measurement method is required.

Accordingly, the primary aim of this study was to evaluate the feasibility and reproducibility of a simplified geometric method for standardized LVT volume assessment by CMR. Secondary analyses exploring associations with LVT persistence at 3 months and 1-year clinical outcomes were hypothesis-generating only.

## Methods

### Study population

This study was a post-hoc methodological analysis of the COVERT-MI trial (COlchicine for left VEntricular Remodeling Treatment in acute Myocardial Infarction; ClinicalTrials.gov identifier: NCT03156816)[28]., a prospective, multicentre, randomized, double-blind, placebo-controlled trial evaluating short-term colchicine therapy after first ST-segment elevation myocardial infarction (STEMI)[28].

In the parent study, 192 adult patients referred for primary percutaneous coronary intervention were enrolled between July 20, 2018 and July 28, 2020. The main trial results and 1-year follow-up have been published previously[29, 30].

For the present analysis, we screened all COVERT-MI participants who underwent the protocol-defined CMR examination at 5 ± 2 days after admission. Among them, patients with left ventricular thrombus (LVT) identified on the baseline CMR constituted the study population for assessment of thrombus measurement reproducibility. Follow-up analyses of thrombus persistence were performed in patients who also underwent the scheduled 3-month CMR examination (3 months ± 15 days). Associations between baseline thrombus measurements and 1-year clinical outcomes were assessed as exploratory secondary analyses.

The primary objective of this post-hoc study was to evaluate the feasibility and reproducibility of a simplified geometric method for standardized CMR quantification of LVT. Prognostic analyses were considered hypothesis-generating only and were not intended to provide definitive evidence regarding predictors of thrombus persistence or clinical events.

The COVERT-MI study protocol was approved by the relevant institutional review board/ethics committee (CPP: 17/084), and all participants provided written informed consent. The present post-hoc analysis was conducted in accordance with the protocol and prior regulatory approvals of the parent study.

### CMR acquisition and analysis

Centralized off-line analysis of all CMR studies was performed on a dedicated workstation (cvi42, Circle Cardiovascular Imaging Inc., Calgary, AB, Canada) by two experienced level-2 observers (CDB and AF), both blinded to clinical characteristics, outcomes, and study status. LV volumes and function were quantified according to standard CMR practice, and infarct size was assessed on LGE images using the full-width half-maximum method.

Reproducibility analyses were performed on the 25 baseline CMR examinations demonstrating LVT and on the 5 follow up CMR of patients with persistent LVT. For intra-reader reproducibility assessment, one observer (AF) repeated all measurements two months later.

All CMR examinations were performed using ECG gating and multi-element phased-array cardiac coils. Imaging protocol included breath-held retrospective ECG-gated steady-state free precession sequences in long- and short-axis of the heart, covering the entire left ventricule.

LGE imaging was performed 10 min after contrast injection of gadolinium (0.2 mmol/kg gadoterique acid, Dotarem^®^, Guerbet, France) using 3D- gradient echo sequences covering the left ventricle in short-axis. Additional two-chamber and four-chamber long-axis 3D-phase-sensitive inversion recovery sequences were also performed to comprehensively assess LGE.

LVT was primarily identified on contrast-enhanced inversion-recovery imaging. No prespecified sequence was mandated for thrombus measurement. In routine practice, the reader first reviewed late gadolinium enhancement (LGE) images and then cine images, and selected the sequence that provided the clearest delineation of thrombus borders in two approximately orthogonal planes for geometric measurements. In the large majority of cases, LGE images were used for measurement, whereas cine images were used only in a minority of cases when thrombus contour appeared better defined. This pragmatic approach was chosen to reflect real-world image interpretation, but sequence selection was not formally standardized and the impact of LGE- versus cine-based measurements was not specifically analyzed.

### Standardized Thrombus Measurement Method

To provide a pragmatic and standardized estimate of LVT burden, we used a simplified geometric method based on three orthogonal thrombus measurements. This approach was selected for feasibility and reproducibility, and was intended as a practical quantitative framework rather than a substitute for planimetric volumetric analysis.

**Fig. 1:**
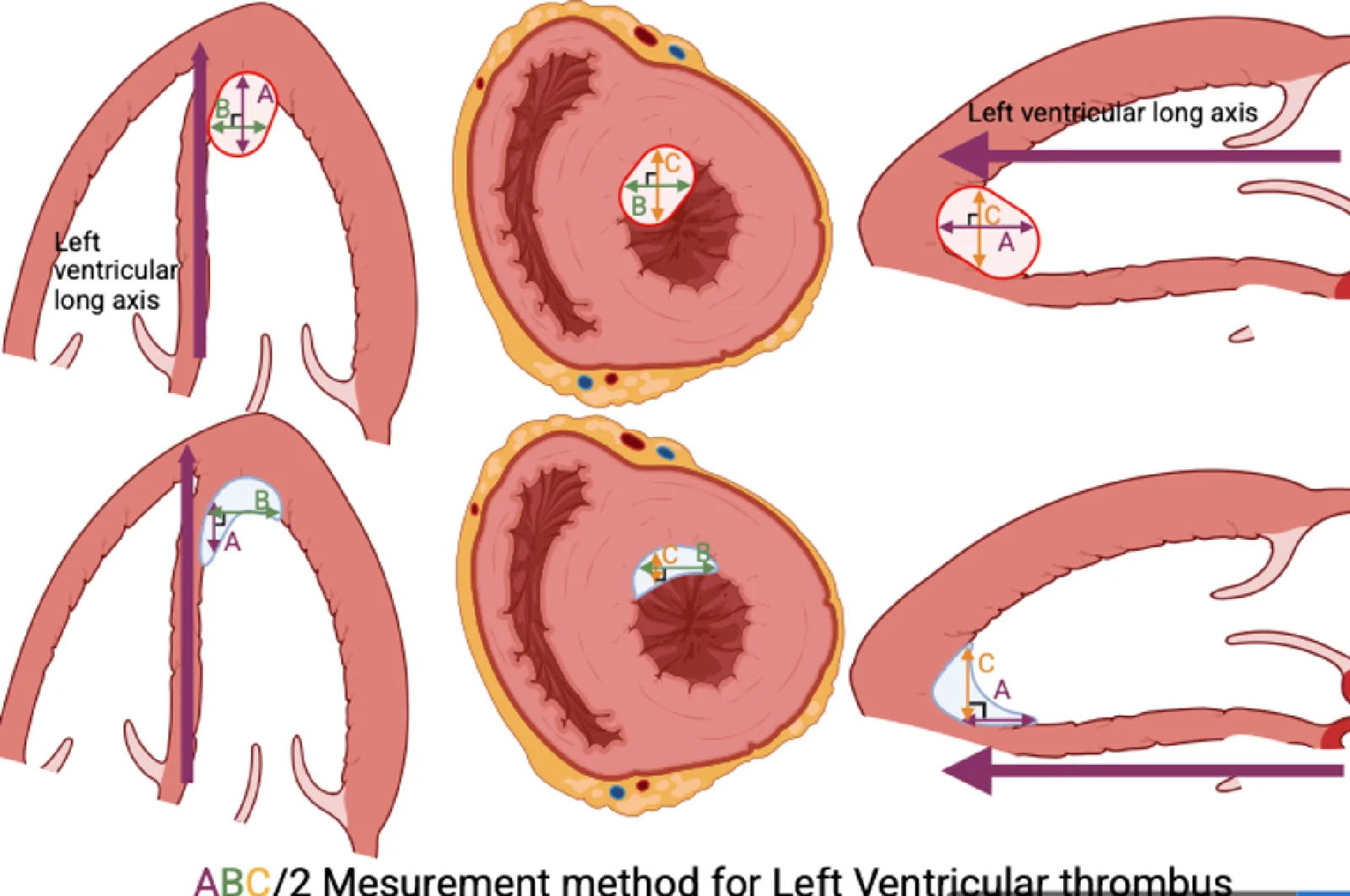
Measurement technique

The following dimensions were obtained from the sequence and views that best delineated thrombus borders: A, maximal thrombus depth along the long axis of the LV; B, maximal width perpendicular to A; and C, maximal height perpendicular to A (Fig. 1). For the primary analysis, thrombus volume was estimated using the ABC/2 formula for all LVTs.

This formula has also been used more recently to evaluate infarct volumes on cerebral Magnetic Resonance Imaging following ischemic stroke[31].

**Fig. 2:**
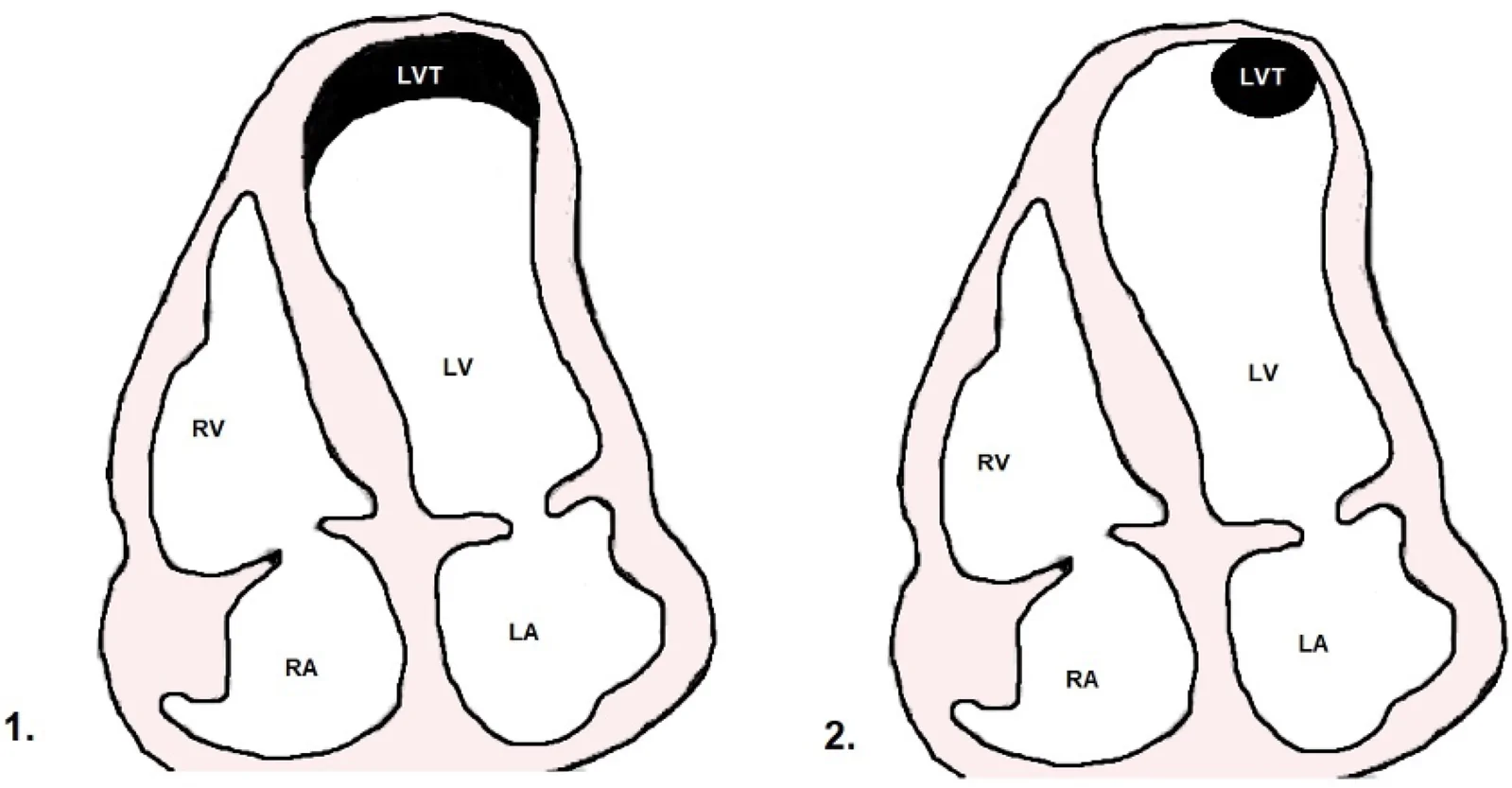
Uni and biconvexe Left Ventricular Thrombus (LVT) 1: Uniconvex LVT (mural) 2: Biconvex LVT (ellipsoid)

Because thrombus morphology may vary, we additionally performed an exploratory shape-adapted analysis (Fig. 2), applying ABC/2 to uniconvex thrombi and ABC × 0.52 to biconvex thrombi, the latter corresponding to a conventional ellipsoid approximation[32–36]. This secondary analysis was designed to assess the influence of gross thrombus shape on estimated volume, not to validate one geometric model over another.

**Fig. 3:**
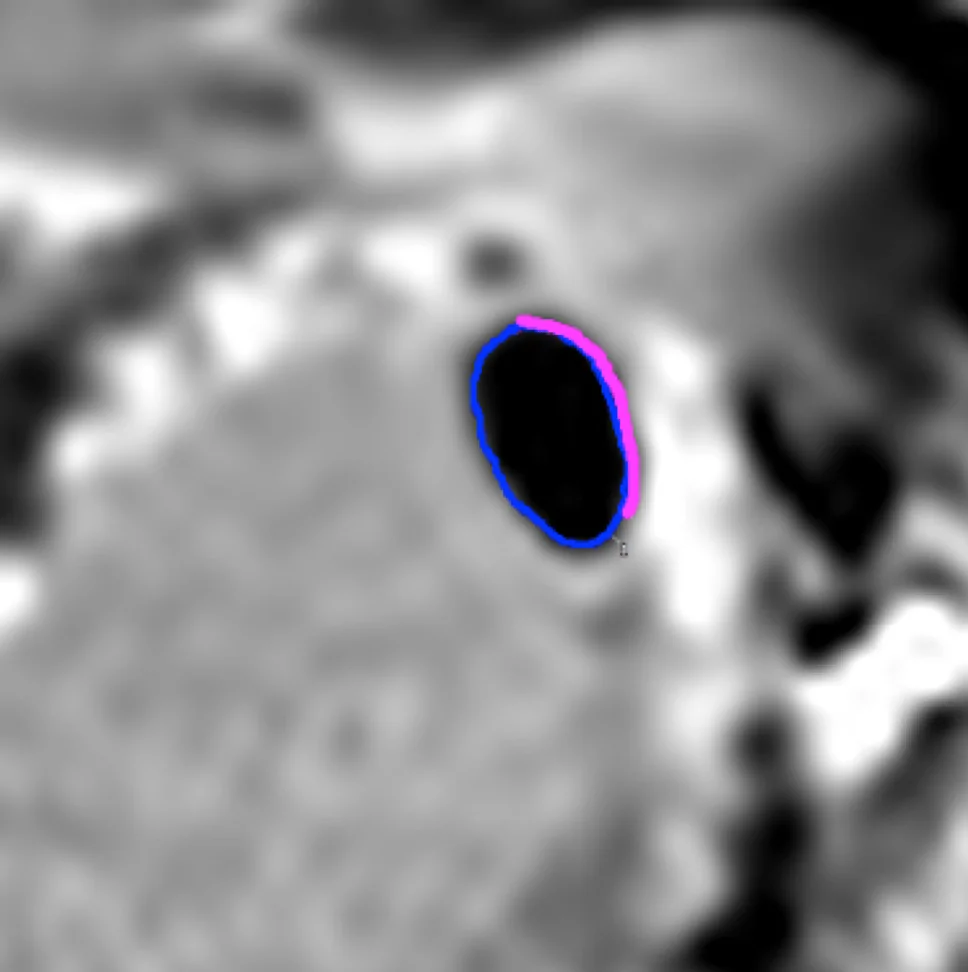
Surface (blue line is the perimeter) and insertion circumference (pink line)

**Fig. 4:**
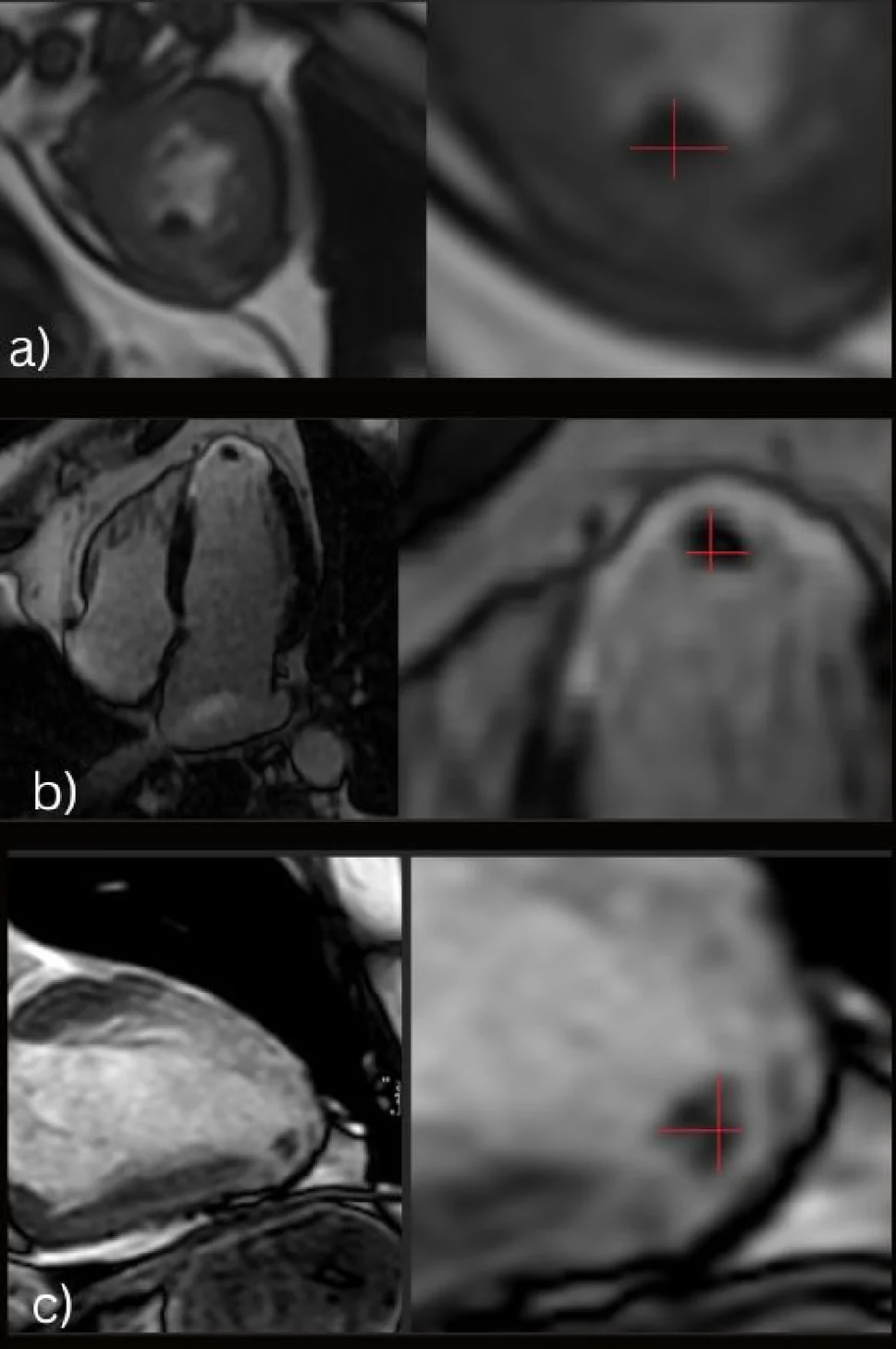
Measurements: (a) Height and width (perpendicular to each other and to the depth) (b) Long axis 4 chambers view: Depth and width (perpendicular to each other and to the height) (c) Long axis 2 chambers view: Height and depth (perpendicular to each other and to the width)

Thrombus area and insertion circumference along the endocardial border were also measured in a standardized manner on the images used for geometric assessment (Figs. 3 and 4).

Thrombus regression at 3 months was defined as complete disappearance on follow-up CMR, with concordant assessment by both readers.

No direct comparison with planimetric CMR-derived thrombus volume was performed.

### Follow-up and late clinical outcomes

The COVERT MI cohort database provided all clinical, outcome, TTE and CMR data (apart from LVT assessment) up to one year of follow-up. All adverse events and MACE were prospectively reported at one year follow-up.

Anticoagulation strategy (vitamin K antagonists or direct oral anticoagulants) and antiplatelet therapy at 3 months were collected and explored as covariates in relation to LVT persistence.

## Statistical analysis

Continuous variables are presented as mean ± standard deviation when normally distributed, or as median [interquartile range] otherwise. Categorical variables are presented as counts and percentages. Comparisons between patients with and without LVT persistence at 3 months were performed using the Chi-square test or Fisher’s exact test for categorical variables, and the Mann–Whitney U test for continuous variables, given the small sample size and non-Gaussian distribution of several variables.

Reproducibility of thrombus shape classification was assessed using Cohen’s kappa coefficient. Inter- and intra-reader agreement for quantitative thrombus measurements was evaluated using Bland–Altman analysis, with calculation of mean bias, limits of agreement, and coefficient of repeatability (also referred to as minimal detectable change). Pearson correlation coefficients and standard error of the estimate (SEE) were additionally calculated to assess agreement between repeated measurements.

All analyses evaluating associations between baseline thrombus characteristics, thrombus persistence at 3 months, and clinical outcomes were considered exploratory and hypothesis-generating only. Given the limited number of patients with LVT and, in particular, the very small number of persistent thrombi, no multivariable modelling was performed and no formal inference regarding predictors of persistence or outcomes was intended. Accordingly, p values from between-group comparisons should be interpreted descriptively and were not used to support claims of association in the absence of statistical significance. All tests were two-sided, and a p value < 0.05 was considered statistically significant for descriptive exploratory purposes. Statistical analyses were performed using Medistica and pvalue.io (Ostend, Belgium).[37].

## Results

### LVT characteristics

Among the 192 patients enrolled in the COVERT-MI trial, 161 underwent the protocol-defined baseline CMR examination at 5 ± 2 days after admission. Left ventricular thrombus (LVT) was identified in 25 patients, who constituted the study population for the present analysis (Table 1).

**Table 1:** Patient characteristics

	LVT persistence (*n* = 5)	LVT regression (*n* = 20)	*p*	Total (*n* = 25)
Age (years)	57.5 (44.8;65.2)	63 (62;68)	0.31	61 (53;66)
Gender male	4 (80%)	17 (85%)	1	21 (84%)
Dyslipidemia	2 (40%)	6 (30%)	1	8 (32%)
Type 2 diabetes	0 (0%)	2 (10%)	1	2 (8%)
Tobacco	3 (60%)	9 (45%)	0.64	12 (48%)
BMI (kg/m²)	26.4 (23.5;28.5)	25.9 (24.8;26.6)	0.53	26.2 (23.5;28.4)
History of coronary heart disease	0 (0%)	0 (0%)	1	0 (0%)
Anticoagulation prior to STEMI	0 (0%)	1 (5%)	1	1 (4%)
Time laps pain/revascularization (min)	190 (155;352)	413 (342;551)	0.18	215 (155;421)
Time laps medical care/revascularization (min)	80 (52;147)	102 (75;121)	0.43	90 (57;146)
Thrombolysis	0 (0%)	5 (25%)	0.54	5 (20%)
Culprit lesion: LAD	5 (100%)	20 (100%)	1	25 (100%)
Admission Creatinine	80.5 (69.2;89.5)	69 (69;71)	0.12	88 (74;96)
TTE LVEDV (ml)	159 (144;194)	180 (159;182)	0.86	162 (145;188)
TTE LVEF (%)	42.5 (35;46.2)	45 (45;46)	0.24	45 (35;46)
CMR LVEF	39,5 (36,6;42,9)	44 (40,6;44)	0,25	40,6 (37,4;44)
CMR infarct size (%)	36.5 (27;42.4)	34.6 (30.5;41.9)	0.97	35.4 (27.7;41.9)
CMR microvascular obstruction weight (g)	4.79 (0;12.5)	2.96 (2;5)	0.81	4.59 (0;10.6)
VKA treatment at 3 month (%)	3 (60%)	8 (44%)	0,64	11 (48%)
AOD treatment at 3 month (%)	0 (0%)	6 (33%)	0,27	6 (26%)

Baseline thrombus characteristics are summarized in Table 2. Using the standardized geometric approach, the mean estimated baseline thrombus volume was 1.03 ± 1.09 cm³, with a median volume of 0.76 cm³ [IQR 0.22–1.29]

**Table 2:** Thrombus shape and regression potential

	LVT regression (*n* = 20)	LVT persistence (*n* = 5)	*p*
Average Height (cm)	1.19 (± 0.38)	1.34 (± 0.705)	0.97
Average Width (cm)	1.2 (± 0.409)	1.23 (± 0.472)	0.91
Average Depth (cm)	1.52 (± 0.667)	1.15 (± 0.422)	0.094
Average Volume (cm^3^)	0.984 (± 0.453)	1.15 (± 1.1)	0.86
Average Insertion circumference A2C (cm)	2.05 (± 0.952)	1.56 (± 0.879)	0.4
Average Insertion circumference A4C (cm)	1.95 (± 0.295)	1.51 (± 0.747)	0.081
Average Surface A2C (cm^2^)	1.06 (± 0.573)	1.16 (± 0.716)	0.97
Average Surface A4C (cm^2^)	0.757 (± 0.305)	1.04 (± 0.556)	0.48

Average was calculated between the measurements of observer 1 and the two measurements of observer 2

The median thrombus insertion circumference along the endocardial border was 1.33 cm [IQR 0.62–2.21], and the median thrombus area was 0.98 cm² [IQR 0.61–1.45].

Regarding thrombus morphology, 19 thrombi (76%) showed a biconvex configuration, whereas 6 thrombi (24%) had a flatter morphology (uniconvex) according to the predefined geometric classification. Most thrombi were located in the apical region of the left ventricle, consistent with the infarct territory in this cohort.

The average thrombus depth (A), width (B), and height (C) measured using the standardized geometric approach are detailed in Table 2.

At the 3-month follow-up CMR examination, thrombus regression was observed in the majority of patients, whereas 5 patients had persistent LVT. Baseline thrombus dimensions were not significantly different between patients with and without thrombus persistence (Table 2). Given the small number of persistent thrombi, these comparisons should be interpreted as exploratory.

During the 1-year follow-up period, no cardioembolic events were recorded in this cohort.

### Measurement technique and reproducibility

Agreement between readers for thrombus volume estimation using the standardized geometric method was high.

Inter-observer reproducibility showed a bias of 0.07 cm³, with limits of agreement ranging from − 0.93 to 1.06 cm³ and a minimal detectable change (MDC) of 1.41 cm³. Inter-observer correlation was high (*r* = 0.86, *p* < 0.001), with a standard error of the estimate (SEE) of 0.51 cm³.

**Fig. 5:**
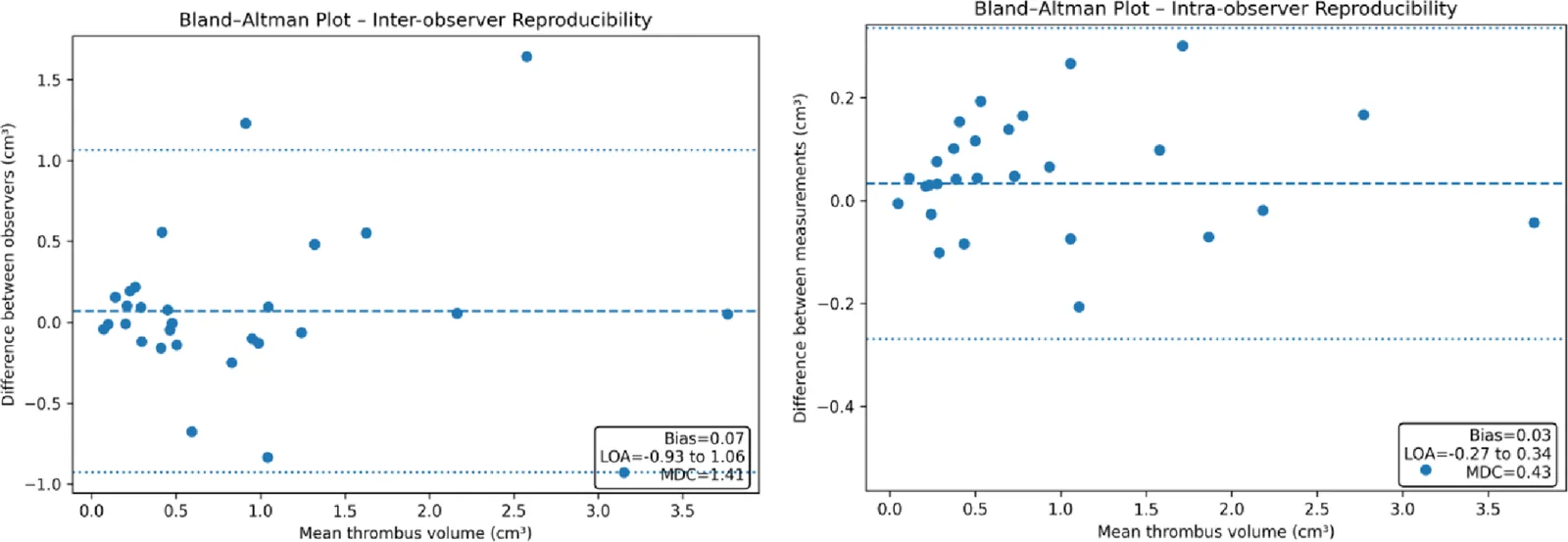
Bland-Altman Plots

Intra-observer reproducibility showed a bias of 0.03 cm³, with limits of agreement ranging from − 0.27 to 0.34 cm³ and a minimal detectable change (MDC) of 0.43 cm³. Intra-observer correlation was excellent (*r* = 0.99, *p* < 0.001), with an SEE of 0.15 cm³ (Table 3; Fig. 5).

**Table 3:** Inter and intra-rater reliability

	Bias (95%)	LLA	ULA	MDC (95%)	Pearson *r*	SEE
Intra-rater	0.03 (−0,13;0,27)	−0.27	0.34	0.43 (0.34; 0.58)	0,99	0,15cm^3^
Inter-rater	0.07 (−0,03;0,09)	−0.93	1,06	1,41 (0.12; 1.89)	0,86	0,51cm^3^

LLA : lower limit of agreement; ULA : upper limits of agreement; MDC : minimal detectable change; SEE: Standard error of the estimate; All measures are in cm^3^

### Shape-dependent measurement

Evaluation of thrombus shape (uniconvex vs. biconvex; mural vs. ellipsoid) showed high inter-reader reproducibility, with a Cohen’s kappa coefficient of 0.90, indicating strong agreement between observers.

In a secondary exploratory analysis accounting for thrombus geometry, the ABC/2 formula was applied to planar (uniconvex) thrombi, whereas the ABC × 0.52 formula was used for ellipsoid (biconvex) thrombi.

The mean difference in estimated volume between the universal ABC/2 approach and the shape-adapted method was 0.035 ± 0.042 cm³, with a maximum absolute difference of 0.15 cm³. The two estimation approaches were strongly correlated (*r* = 0.98, *p* < 0.001).

The theoretical difference between the two geometric formulas (approximately 4%) translated into a very small absolute difference in thrombus volume (< 0.15 cm³), which remained well below the minimal detectable change observed for thrombus volume measurements.

### Factors associated with LVT persistence

No baseline thrombus dimension, including thrombus height, width, or depth, was significantly associated with thrombus persistence at three months (Table 2). Similarly, thrombus surface area and insertion circumference were not significantly associated with persistence.

Thrombus morphology was also not significantly associated with thrombus persistence. Persistent thrombus was observed in 3 of 6 uniconvex (mural) thrombi (50%) and in 2 of 19 biconvex (ellipsoid) thrombi (10%), although this comparison was based on very small subgroup counts.

LVT persistence was not significantly associated with the presence of anticoagulation therapy (vitamin K antagonists or direct oral anticoagulants) at three months. Curative anticoagulation was present in 14 patients (78%) with thrombus regression and 3 patients (60%) with persistent thrombus (*p* = 0.58).

Baseline left ventricular ejection fraction was also not significantly different between patients with thrombus regression and those with persistent thrombus (median 44% [40–44] vs. 39.5% [37–42], *p* = 0.25).

Given the very small number of persistent thrombi, all these analyses should be considered exploratory.

### Impact of LVT measurement on adverse clinical outcomes

In this cohort of 25 patients with LVT, no cardioembolic events, including ischemic stroke, were recorded during the 1-year follow-up.

As a result, it was not possible to evaluate the relationship between baseline thrombus measurements and adverse clinical outcomes. The absence of events precluded any meaningful assessment of the potential prognostic value of LVT volume, morphology, or other thrombus characteristics in this cohort.

## Discussion

The present study evaluated a simplified geometric method for estimating left ventricular thrombus (LVT) volume using cardiac magnetic resonance (CMR). The main findings are threefold. First, a standardized geometric approach based on three orthogonal measurements provided high inter- and intra-reader reproducibility for estimating thrombus volume. Second, accounting for thrombus shape had minimal impact on volume estimation, suggesting that a universal ABC/2 approximation may be sufficient for practical use in thrombi with relatively simple morphology. Third, in this cohort, no thrombus characteristic was significantly associated with thrombus persistence at 3 months, and no cardioembolic events occurred during the one-year follow-up. Accordingly, this study should be interpreted primarily as a methodological and reproducibility study, rather than a prognostic investigation.

### Standardized geometric assessment of LVT volume

Quantification of LVT burden has been previously performed using planimetric techniques on contrast-enhanced CMR images. Prior studies by Weinsaft and colleagues[12, 23–25] demonstrated the ability of delayed enhancement CMR to detect small and mural thrombi and reported planimetric thrombus volume measurements. Similarly, Velangi et al.[21] assessed thrombus volume by CMR planimetry and evaluated long-term embolic outcomes in a substantially larger cohort, while Bawaskar et al.[38] recently reported the evolution of LVT using serial CMR examinations.

In this context, the aim of the present study was not to replace planimetric quantification but to evaluate the feasibility and reproducibility of a simplified geometric approach for estimating thrombus volume. Such an approach may be particularly useful when rapid, standardized measurements are required, for example in multicenter datasets or longitudinal follow-up studies. The ABC/2 formula has been widely used in other areas of medical imaging as a pragmatic geometric approximation for estimating lesion volume. In the present study, this simplified method showed high reproducibility with minimal inter- and intra-reader bias, suggesting that it can provide a consistent estimate of thrombus burden when applied to CMR images.

Bland–Altman analysis demonstrated limited systematic bias, while Pearson correlation and SEE confirmed a strong linear relationship between repeated measurements, particularly for intra-reader assessment. However, the wider limits of agreement and higher minimal detectable change observed for inter-reader measurements highlight that this approach, although reproducible, remains partly dependent on reader interpretation and image plane selection. Therefore, standardized acquisition planes, clear measurement rules, and reader training remain important when applying this method in longitudinal or multicenter studies.

Importantly, our findings address measurement reproducibility rather than volumetric accuracy. Because no direct comparison with planimetric CMR-derived volumes was available, the present study should not be interpreted as validating the ABC/2 method against established volumetric techniques. Rather, they demonstrate that a simple geometric framework can provide consistent and reproducible measurements of LVT volume.

### Influence of thrombus morphology on volume estimation

Thrombus morphology varies across patients and may theoretically influence geometric volume estimation. In our cohort, thrombi were predominantly located in the apical region and generally displayed relatively simple shapes. In the exploratory shape-adapted analysis, the difference between the universal ABC/2 approach and a shape-adjusted formula (ABC × 0.52 for ellipsoid thrombi) was very small in absolute terms and remained well below the minimal detectable change for thrombus volume measurements.

These findings suggest that, in thrombi with relatively simple geometry, a universal ABC/2 approximation may be sufficient for practical use. However, this observation should be interpreted cautiously, as more complex thrombus shapes were uncommon in the present cohort. Larger studies including more diverse thrombus morphologies would be required to determine whether shape-specific geometric models provide additional benefit.

### LVT persistence and clinical outcomes

In the present cohort, thrombus regression was observed in the majority of patients at 3 months. None of the thrombus characteristics measured in this study, including thrombus dimensions, volume, morphology, surface area, or insertion circumference, was significantly associated with thrombus persistence.

Persistent thrombi were numerically more frequent among uniconvex thrombi, but this observation was based on very small subgroup counts and should not be interpreted as evidence of an association. Similarly, the absence of cardioembolic events during follow-up prevented any meaningful assessment of the relationship between LVT measurements and clinical outcomes.

These results reinforce the methodological nature of the present study. The absence of statistically significant associations should not be interpreted as evidence that thrombus size or morphology has no clinical relevance, but rather as a consequence of limited statistical power and low event rates. Larger cohorts will be required to determine whether standardized quantitative LVT assessment may improve prediction of thrombus persistence, embolic risk, or treatment response.

### Study limitations

Several limitations should be acknowledged. First, the sample size was relatively small, particularly regarding the number of patients with LVT and the number of persistent thrombi at 3 months. This limited statistical power, precluded multivariable analyses, and prevented any robust assessment of predictors of thrombus persistence.

Second, no cardioembolic events occurred during follow-up in patients with LVT. Consequently, the present study cannot determine whether LVT volume, shape, surface area, or insertion circumference are associated with embolic risk or clinical outcomes. These analyses should therefore be considered exploratory and hypothesis-generating only.

Third, scan–rescan reproducibility was not assessed. This is an important limitation because geometric measurements may be influenced by image plane prescription, especially for small or irregular thrombi. Future studies using standardized or isotropic three-dimensional acquisitions may help determine the robustness of this method across repeated examinations.

Fourth, sequence selection for thrombus measurement was not prespecified. Although measurements were performed on the images providing the clearest thrombus delineation, this pragmatic approach may have introduced heterogeneity. The impact of LGE-, EGE-, or cine-based measurements was not specifically evaluated.

Fifth, no external volumetric reference standard was available. Therefore, this study evaluates reproducibility rather than absolute volumetric accuracy. The ABC/2 method should not be interpreted as validated against planimetric CMR-derived volumes, computed tomography, or pathological measurements.

Finally, the study population was derived from a post-hoc analysis of the COVERT-MI trial and may not reflect the full spectrum of LVT morphology encountered in routine practice. Most thrombi were relatively small and apical, and more complex morphologies were uncommon. Larger and more diverse cohorts will be required to confirm the generalizability of this approach.

### Clinical Implications

Standardized and reproducible quantification of thrombus burden is an important prerequisite for studies investigating LVT evolution and treatment response. The simplified geometric approach evaluated in this study provides a practical framework for estimating LVT volume on routinely acquired CMR images.

Although this method is not intended to replace planimetric volumetric analysis or to guide treatment decisions in isolation, it may facilitate a more consistent description of thrombus burden across readers, centers, and serial examinations. This is particularly relevant in post-myocardial infarction patients, in whom follow-up imaging is commonly used to document thrombus regression but where no standardized quantitative framework is currently available.

In clinical research, this approach may help harmonize LVT assessment in multicenter datasets and future studies evaluating anticoagulation strategies, thrombus regression, or imaging markers of embolic risk. By providing a simple and reproducible measurement framework, the ABC/2 method may also support the development and validation of more advanced imaging approaches, including semi-automated or automated thrombus quantification.

The present findings should therefore be interpreted as establishing a methodological basis for future prognostic and therapeutic studies, rather than as evidence that LVT volume or morphology should currently modify patient management.

Future larger-scale studies will be required to determine whether quantitative thrombus characteristics contribute to predicting thrombus persistence or embolic risk and whether treatment strategies guided by thrombus characteristics may improve clinical outcomes[39].

## Conclusion

In this post-hoc CMR analysis, a simplified geometric approach based on the ABC/2 formula provided a feasible and reproducible method for estimating left ventricular thrombus volume. This approach may offer a practical framework for standardized thrombus quantification in research settings and longitudinal imaging studies.

In this cohort, no thrombus characteristic was significantly associated with thrombus persistence at 3 months, and no cardioembolic events occurred during follow-up. These findings should be interpreted as exploratory given the limited sample size and event rate.

Further studies in larger and more diverse populations, ideally including standardized three-dimensional acquisitions and comparisons with established volumetric techniques, are required to determine the clinical and prognostic relevance of quantitative LVT assessment.

## Data Availability

No datasets were generated or analysed during the current study.

## Abbreviations

CMR: Cardiac Magnetic Resonance
LVT: Left ventricular thrombus
MACE: Major Adverse Cardiovascular Events
MRI: Magnetic Resonance Imaging
SDH: Subdural hematomas
STEMI: ST-elevation myocardial infarction
